# The complete chloroplast genome sequence of the *Alstroemeria* hybrid variety ‘Hanhera’

**DOI:** 10.1080/23802359.2020.1840936

**Published:** 2020-12-24

**Authors:** Jong-Bo Kim, Hwan-Rae Yang, Sang-Hee Lee, Tae-Ho Park

**Affiliations:** a Department of Biotechnology, Research Institute for Biomedical & Health Sciences, College of Biomedical & Health Sciences, Glocal Campus, Konkuk University, Chungju, South Korea; b Department of Horticulture, Daegu University, Gyeongsan, South Korea

**Keywords:** Chloroplast, genome, genome sequence, *Alstroemeria* hybrid

## Abstract

*Alstroemeria*, a member of the Alstroemeriaceae family, is a species from South America. The chloroplast genome of *Alstroemeria* spp. was completed by *de novo* assembly using a small amount of whole genome sequencing data. The chloroplast genome of *Alstroemeria* spp. was 155,672 bp in length consisting of 84,379 bp of large single copy, 17,815 bp of small single copy, and 26,739 bp of a pair of inverted repeat regions. A total of 157 genes were annotated including 103 protein-coding genes (PCGs), 46 *tRNA* genes, and eight *rRNA* genes. Maximum likelihood phylogenetic analysis with seven species belonging to the Alstroemeriaceae or Liliaceae family revealed that *Alstroemeria* spp. is grouped with the species in the Alstroemeriaceae family.

*Alstroemeria* belonging to the Alstroemeriaceae family is a popular cut flower plant because of the wide variety of flower colors and includes approximately 60 species (Aker and Healy 1990). The native species are found with many different shades of reds and yellow colors in numerous habitats and the wide color range in the interspecific hybrids arises from this diverse color base found in the species. The white and yellow types of color originated from one interspecific cross and the red and orange types of color originated from several different interspecific crosses (Healy and Wilkins 1986). Interspecific breeding is considered as the most important source of genetic variation in ornamental crops including *Alstroemeria*. However, albino progenies and variegated-color phenotypes caused by plastome-genome incompatibility appear during interspecific breeding in diverse plants, such as the genus, *Pelargonium* (Metzlaff et al. 1982), *Trifolium* (Pandey et al. 1987), *Zantedeschia* (Yao et al. 1994, 1995), etc. Therefore, the information of chloroplast genome of the hybrid ‘Hanhera’ (*Alstroemeria* spp.) obtained in this study will provide an insight of plastid inheritance, investigate more detailed evolutionary aspect, and facilitate breeding of colored *Alstroemeria* efficiently.

The *Alstroemeria* variety ‘Hanhera’ was provided by Prof. Tae-Ho Han, Department of Horticulture, Chonnam National University, South Korea (35.2° N, 126.9° E). It is an *Alstroemeria* hybrid (*Alstroemeria* spp.) derived from a cross between other *Alstroemeria* spp. hybrids ‘Leon’ and ‘Everest’. Their genetic backgrounds are complex because they are originally derived from interspecific crosses between different species belonging to the *Alstroemeria* genus. The variety ‘Handera’ has white-colored flowers with very faint pink and yellow petal. Its flower size is bigger and plant height is taller than other *Alstroemeria* varieties. The completion of the chloroplast genome sequence was performed at Phyzen bioinformatics pipeline (Kim et al. 2015). According to the PE standard protocol (Illumina, San Diego, CA), an Illumina paired-end (PE) genomic library was constructed with a total genomic DNA and they were sequenced using an Illumina HiSeq2000 platform at Macrogen (http://www.macrogen.com/kor/). The chloroplast genome assembly was performed with approximately 1.9 Gbp of trimmed high-quality reads obtained by a CLC assembly cell package version 4.2.1 (CLC Inc., Aarhus, Denmark). The principal contigs representing the chloroplast genome were retrieved using Nucmer (Kurtz et al. 2004) with the chloroplast genome sequence of *Alstroemeria aurea* (KC968976, Do et al. 2013; Kim and Kim 2013) as reference sequence. The representative chloroplast contigs were arranged in order based on BLASTZ analysis (Schwartz et al. 2003) and connected to a single draft sequence by joining overlapping terminal sequences and manual editing through a comparison with the reference chloroplast genome sequence of *Alstroemeria aurea* (KC968976) as described previously (Cho et al. 2015; Cho and Park 2016). GeSeq program (Tillich et al. 2017) and manual curation based on the results of BLAST searches were used for gene annotation.

The complete chloroplast genome of the *Alstroemeria* hybrid variety ‘Hanhera’ (GenBank accession no. MK327552) was 155,672 bp in length including 26,739 bp inverted repeats (IRa and IRb) regions separated by small single copy (SSC) region of 17,815 bp and large single copy (LSC) region of 84,379 bp. When the chloroplast genome sequence was compared with those of other Alstroemeriaceae species, such as *A. aurea* (KC968976, Do et al. 2013; Kim and Kim 2013) and *Bomarea edulis* (KM233641, Kim et al. 2016), it was slightly longer and the sequence conservation between them was expectedly very high with a sequence identity of 99 and 96%, respectively. The structure was quadripartite that is typical in most plastids, and the structure and gene features were typically identical to those of higher plants. A total of 157 genes with an average size of 588.2 bp were annotated including 103 protein-coding genes (PCGs) with an average size of 780.9 bp, 46 *tRNA* genes, and eight *rRNA* genes with an average size of 220.7 bp. An overall GC content was 38.05%.

The results from the phylogenetic analysis performed using chloroplast coding sequences of the *Alstroemeria* hybrid ‘Hanhera’ and three and four published species in the Alstroemeriaceae and Liliaceae family, respectively, by a maximum likelihood method in MEGA version 6.0 (Tamura et al. 2013) revealed that the *Alstroemeria* hybrid ‘Hanhera’ was expectedly grouped with *A. aurea* followed by *Bomarea edulis* and *Luzuriaga radicans* which all belonged to the Alstroemeriaceae family (Figure 1).

**Figure 1.**
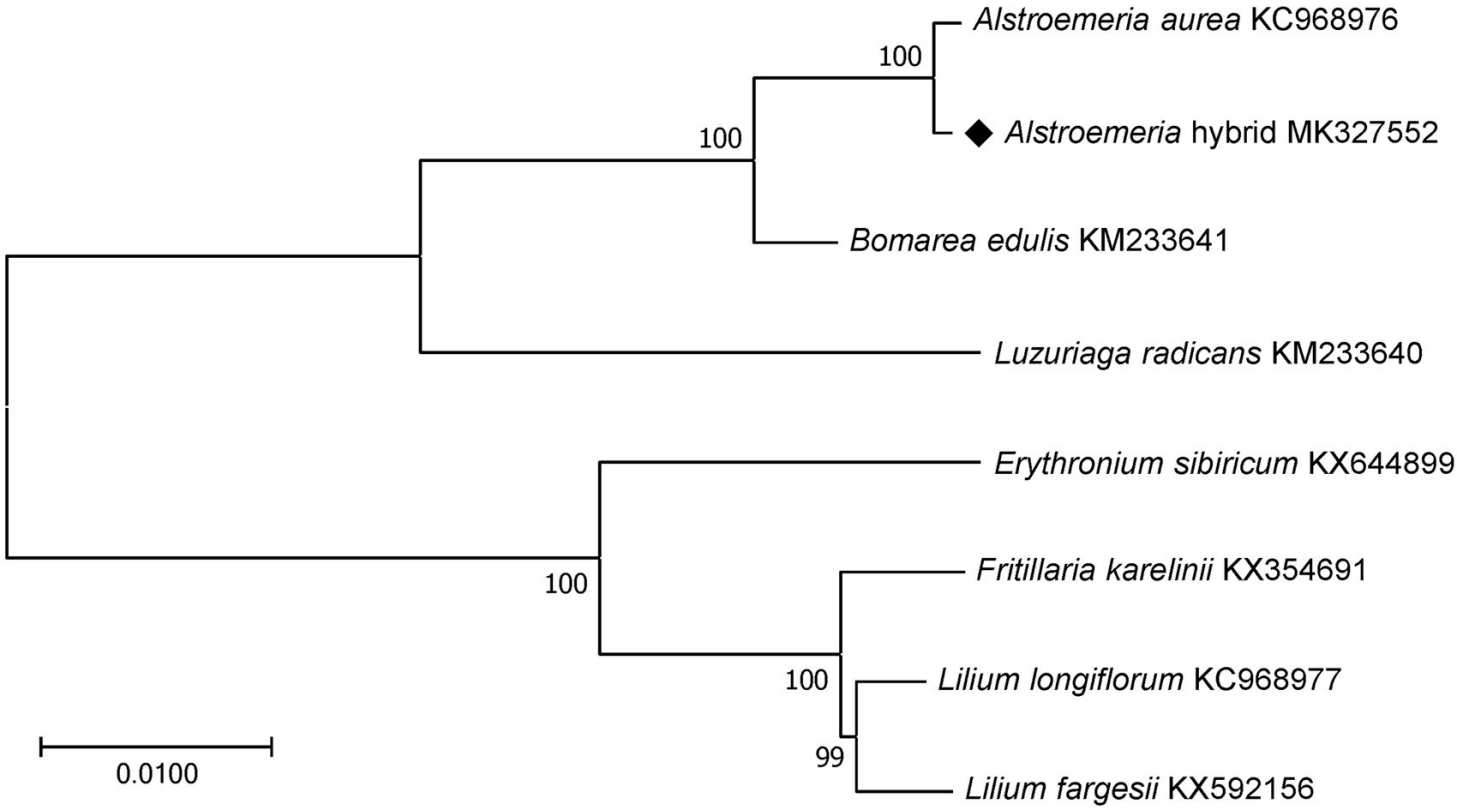
Maximum likelihood phylogenetic tree of the *Alstroemeria* hybrid ‘Hanhera’ with seven species belonging to the Alstroemeriaceae or Liliaceae based on chloroplast protein-coding sequences. Numbers in the nodes are the bootstrap values from 1000 replicates.

## Data Availability

The data that support the findings of this study are openly available in GenBank of NCBI at https://www.ncbi.nlm.nih.gov, reference number MK327552.

## References

[CIT0001] Aker S, Healy W. 1990. The phytogeography of the genus *Alstroemeria*. Herbertia. 46:76–87.

[CIT0002] Cho KS, Park TH. 2016. Complete chloroplast genome sequence of *Solanum nigrum* and development of markers for the discrimination of *S. nigrum*. Hortic Environ Biotechnol. 57(1):69–78.

[CIT0003] Cho KS, Yun BK, Yoon YH, Hong SY, Mekapogu M, Kim KH, Yang TJ. 2015. Complete chloroplast genome sequence of tartary buckwheat (*Fagopyrum tataricum*) and comparative analysis with common buckwheat (*F. esculentum*). PLOS One. 10(5):e0125332. 25966355 10.1371/journal.pone.0125332 PMC4428892

[CIT0004] Do HDK, Kim JS, Kim JH. 2013. Comparative genomics of four Liliales families inferred from the complete chloroplast genome sequence of *Veratrum patulum* O. Loes. (Melanthiaceae). Gene. 530(2):229–235. 23973725 10.1016/j.gene.2013.07.100

[CIT0005] Healy WE, Wilkins HF. 1986. Alstroemeria culture. Herbertia. 42:16–20.

[CIT0006] Kim JS, Kim HT, Yoon CY, Kim JH. 2016. The complete plastid genome sequence of *Bomarea edulis* (Alstroemeriaceae: Liliales). Mitochondrial DNA Part A. 27:1869–1870. 10.3109/19401736.2014.971264 25319309

[CIT0007] Kim JS, Kim JH. 2013. Comparative genome analysis and phylogenetic relationship of order Liliales insight from the complete plastid genome sequences of two Lilies (*Lilium longiflorum* and *Alstroemeria aurea*). PLoS One. 8(6):e68180. 23950788 10.1371/journal.pone.0068180 PMC3688979

[CIT0008] Kim K, Lee SC, Lee J, Yu Y, Yang K, Choi BS, Koh HJ, Waminal NE, Choi HI, Kim NH, et al. 2015. Complete chloroplast and ribosomal sequences for 30 accessions elucidate evolution of *Oryza* AA genome species. Sci Rep. 5:15655. 26506948 10.1038/srep15655 PMC4623524

[CIT0009] Kurtz S, Phillippy A, Delcher AL, Smoot M, Shumway M, Antonescu C, Salzberg SL. 2004. Versatile and open software for comparing large genomes. Genome Biol. 5(2):R12. 14759262 10.1186/gb-2004-5-2-r12 PMC395750

[CIT0010] Metzlaf M, Pohlheim F, Börner T, Hagemann R. 1982. Hybrid variegation in the genus *Pelargonium*. Curr Genet. 5(3):245–249. 24186302 10.1007/BF00391813

[CIT0011] Pandey KK, Grant JE, Williams EG. 1987. Interspecific hybridaisation between *Trifolium repens* and *T. uniflorum*. Aust J Bot. 35(2):171–182.

[CIT0012] Schwartz S, Kent WJ, Smit A, Zhang Z, Baertsch R, Hardison RC, Haussler D, Miller W. 2003. Human-mouse alignments with BLASTZ. Genome Res. 13(1):103–107. 12529312 10.1101/gr.809403 PMC430961

[CIT0013] Tamura K, STecher G, Peterson D, Filipski A, Kumar S. 2013. MEGA6: molecular evolutionary genetics analysis version 6.0. Mol Biol Evol. 30(12):2725–2729. 24132122 10.1093/molbev/mst197 PMC3840312

[CIT0014] Tillich M, Lehwark P, Pellizzer T, Ulbricht-Jones ES, Fischer A, Bock R, Greiner S. 2017. GeSeq – versatile and accurate annotation of organelle genomes. Nucleic Acids Res. 45(W1):W6–W11. 28486635 10.1093/nar/gkx391 PMC5570176

[CIT0015] Yao JL, Cohen D, Rowland RE. 1994. Plastid DNA inheritance and plastome-genome incompatibility in interspecific hybrids of *Zantedeschia* (Araceae). Theor Appl Genet. 88(2):255–260. 24185935 10.1007/BF00225906

[CIT0016] Yao JL, Cohen D, Rowland RE. 1995. Interspecific albino and variegated hybrids in the genus *Zantedeschia*. Plant Sci. 109(2):199–206.

